# Expression of Tissue Inhibitors of Metalloproteinases (TIMP-1, TIMP-2, TIMP-3, TIMP-4) in Blood Serum of Patients with Keratoconus

**DOI:** 10.3390/jcm13041168

**Published:** 2024-02-19

**Authors:** Marta Nowak-Wąs, Paweł Wąs, Zenon Czuba, Romuald Wojnicz, Dorota Wyględowska-Promieńska

**Affiliations:** 1 Department of Histology and Cell Pathology, Faculty of Medical Sciences in Zabrze, Medical University of Silesia, 40-055 Katowice, Poland; 2 Department of Ophthalmology, Kornel Gibinski University Clinical Center, Medical University of Silesia, 40-055 Katowice, Poland; 3 Department of Ophthalmology, Megrez Provincial Specialist Hospital in Tychy, 43-100 Tychy, Poland; 4 Department of Microbiology and Immunology, Faculty of Medical Sciences in Zabrze, Medical University of Silesia, 40-055 Katowice, Poland; 5 Department of Ophthalmology, Faculty of Medical Sciences in Katowice, Medical University of Silesia, 40-055 Katowice, Poland

**Keywords:** keratoconus, cornea, tissue inhibitors of matrix metalloproteinases

## Abstract

Background: The etiology of keratoconus is unclear. Current evidence suggests that inflammatory and systemic mechanisms might play a role in its pathophysiology. The proper interaction of proteolytic enzymes—matrix metalloproteinases—and their specific tissue inhibitors (TIMPs) within the cornea is essential in maintaining its structure, transparency and healing processes. The aim of the study was to determine the concentration of the TIMPs TIMP-1, TIMP-2, TIMP-3, and TIMP-4 in the blood serum samples of patients with keratoconus compared to the control group. Methods: The study encompassed 132 patients, of which 83 people constituted the study group and 49 the control group. The concentration of selected TIMPs was determined using the Human Magnetic Luminex^®^ Performance Assay method. Results: In the study group, the concentrations of TIMP-1 and TIMP-3 were statistically significantly reduced, and TIMP-2 and TIMP-4 increased compared to the control group. The analysis of individual TIMPs in terms of their usefulness as potential predictors of keratoconus showed high results of diagnostic sensitivity and specificity for all TIMPs, in particular for TIMP-1 and TIMP-2. Conclusion: The above results may indicate systemic disturbances in the TIMPs regulation among keratoconus patients. High diagnostic sensitivity and specificity of all TIMPs, in particular TIMP-1 and TIMP-2, may confirm their participation in the etiopathogenesis of this disease.

## 1. Introduction

Keratoconus (KC) is a bilateral, asymmetric disease involving the progressive thinning and steepening of the central and paracentral part of the cornea, which takes the shape of a cone. The result of these changes is a deterioration of vision due to increasing irregular astigmatism and myopia. KC occurs in all ethnic groups and affects both sexes [1]. Keratoconus is a multifactorial disease of unclear etiology and a difficult-to-predict cause. Factors predisposing the occurrence and progression of KC include genetic and environmental causes, e.g., wearing contact lenses, rubbing eyes, atopy, allergy, exposure to ultraviolet radiation or other factors triggering oxidative stress within the cornea. It occurs in an isolated form or is associated with systemic diseases and syndromes, such as Ehlers–Danslos syndrome, Down syndrome, Marfan syndrome or osteogenesis imperfecta [2,3]. According to the traditional definition, keratoconus is a non-inflammatory disease, but current evidence suggests an important role of inflammatory mechanisms [4].

Tissue inhibitors of matrix metalloproteinases (TIMPs) TIMP-1, TIMP-2, TIMP-3, and TIMP-4 are specific endogenous protease inhibitors of matrix metalloproteinases (MMPs). TIMPs are important regulators of extracellular matrix turnover, tissue remodeling, and cell behavior. A disturbance of the balance between MMPs and TIMPs is associated with abnormal extracellular matrix (ECM) degradation and consequently the pathophysiology and progression of several diseases [5].All TIMPs are able to inhibit all known matrix metalloproteinases through reversible blockade, forming 1:1 stoichiometric complexes. However, the inhibition efficiency of MMPs varies depending on the TIMP. TIMPs also selectively inhibit other proteins—such as proteins belonging to the ADAM (a disintegrin and metalloproteinase domain) and ADAMTS (a disintegrin and metalloproteinase with thrombospondin motifs) families. TIMPs are also important for the activation and uptake/removal of MMPs from the extracellular environment [6].TIMPs also have functions independent of MMPs inhibition. They directly bind to receptors on the cell surface. They are expressed by various cell types and are present in most human tissues and body fluids. In specific tissues, TIMPs are expressed constitutively or in an inducing way and are regulated at the transcriptional level by cytokines, growth factors and chemokines [7]. TIMPs regulate ECM turnover in two ways, which depend on the specific metalloproteinase inhibited by a specific TIMP in a particular tissue. On the one hand, they inhibit ECM proteolysis directly by interacting with MMPs and, on the other hand, indirectly, by controlling cell functions (e.g., regulation of inflammation, control of the release of TGFβ1 activation), and they are able to limit ECM deposition or fibrosis [6].

The development of keratoconus is the result of a combination of simultaneous corneal destruction and healing processes and includes cell death, migration, proliferation, differentiation and extracellular matrix remodeling [8]. Matrix metalloproteinases play a key role in corneal healing. Numerous independent studies have demonstrated altered expression of MMPs within the corneal layers and/or tear film of patients with KC, especially MMP-1, MMP-2, MMP-3, MMP-7, MMP-9, and MMP-13 as well as specific TIMP-1, TIMP-2, and TIMP-3 inhibitors [9,10]. TIMPs tend to neutralize the activity of MMPs and thus help maintain ECM stability. Together, they constitute a complex responsible for the integrity of connective tissue and proper healing of wounds after injuries. The proper composition and tight organization of the stromal extracellular matrix is essential in maintaining its transparency, mechanical strength, and stability, including the ability to preserve normal shape and curvature [11]. Imbalances in this process may lead to progressive weakening of the cornea. An imbalance in favor of MMPs may lead to increased tissue degradation and, in contrast, in favor of TIMPs, to the accumulation of extracellular matrixes. It may also promote the initiation of fibrosis leading to tissue remodeling [12].

Recent scientific reports have suggested that systemic factors may play a role in the development of keratoconus. Patients with KC show significantly increased total oxidative status (TOS) and oxidative stress index (OSI) compared to the control group [13]. Moreover, patients with KC showed significantly increased values of inflammatory markers such as the platelet-to-lymphocyte ratio (PLR) and neutrophile-to-lymphocyte ratio (NLR), and increased NLR values were observed in patients with progressive keratoconus compared to stable forms and the control group [14,15]. Studies conducted by Sobrino et al. showed an increased expression of toll-like receptors (TLRs), in particular TLR2 and TLR4, in the blood of patients with keratoconus, which was associated with increased levels of inflammatory mediators in serum, such as nuclear transcription factor NF-kB, IL-6, IL-1B, TNF-α, and MMP-9 [16].

So far, the available literature presents results regarding the expression of selected inhibitors in the cornea and tear film, without correlating the obtained results with the concentration of these proteins in the peripheral blood. The above-mentioned examples indicate the necessity of linking the disease entity of keratoconus with general parameters. According to the available knowledge, this work is the first study that evaluates matrix metalloproteinase inhibitors in blood serum in patients with keratoconus.

## 2. Materials and Methods

### 2.1. Purpose of the Study

The aim of the study was to assess the concentration of selected tissue inhibitors of matrix metalloproteinases TIMP-1, TIMP-2, TIMP-3, and TIMP-4 in the blood serum of patients diagnosed with keratoconus compared to people from the control group.

The Ethics Committee of the Medical University of Silesia, Katowice, Poland, approved the study protocol (approval number: PCN/CDN/0022/KB1/66/21, date of approval: 6 July 2021). Informed consent was signed by all participants. This study complied with the tenets of the Declaration of Helsinki.

### 2.2. Study Population

The analyzed group included a total of 132 people classified into the study or control group, respectively. The study group included 83 patients (21 women and 62 men) with clinically confirmed stable keratoconus (the patients had not exhibited any changes in corneal tomographic parameters over a period of 6 months) in both eyes, the diagnosis of which was based on anclinical image obtained during a slit-lamp examination and corneal tomographic examination performed with the Swept Source anterior segment OCT (AS-OCT) CASIA2 device (Tomey Corporation, Nagoya, Japan). The control group consisted of 49 people, including 29 women and 20 men without corneal pathology and any other ophthalmological diseases. The median age and interquartile ranges in the study and control groups were 35(26.42) and 30(29.34) years, respectively, *p* = 0.34.

### 2.3. Exclusion Criteria from the Study

History of eye trauma and/or suspicion of post-traumatic ectasia; patients after previous refractive surgery or suspected ectasia related to the previous procedure; previous intraocular procedures; previous or active corneal inflammation or ulceration regardless of etiology; corneal neovascularization; pterygium; comorbidities, including diabetes mellitus, hypothyroidism, allergy, cancer, and autoimmune diseases; patients under treatment with systemic anti-inflammatory drugs or antibiotics from the tetracycline group, in particular, doxycycline; and pregnancy or lactation.

Venous blood samples with a volume of 8 mL for laboratory tests were collected in the morning into BD Vacutainer^®^ SST II Advance tubes. After centrifuging 1000× *g* for 15 min, the serum was stored at a temperature below −80°C until biochemical determinations were performed.

TIMP-1, TIMP-2, TIMP-3, and TIMP-4 concentrations were determined through the use of the Human TIMP Magnetic Luminex Performance Assay 4-plex Kit (R&D Systems Inc., Bio-Techne, Minneapolis, MN, USA), catalog number: LKTM003, according to the manufacturer’s instructions.

### 2.4. Statistical Analysis

Data were presented as the median value with the first and third quartiles due to significant deviations from the normal distribution of variables, assessed using the Shapiro–Wilk test. The Mann–Whitney U-test was used to compare quantitative variables between the control and study groups, and the Pearson coefficient was used to assess the correlation between variables. To evaluate selected variables as potential predictors, a generalized linear model with a logit binding function (logistic regression model) was used. The results of univariate and multivariate analyses were presented as ROC curves with specificity sensitivity, a positive and negative predictive value and AUC (area under curve).

In order to avoid the phenomenon of multicollinearity, factors obtained from the selected variables using the principal components method were introduced into the multifactor model. The analysis was performed using the R language in the RStudio environment using the pROC, factoextra, fviz and tidyverse libraries. *p* values less than 0.05 were considered significant.

## 3. Results

The median concentration of all TIMPs between groups differed in a significant manner (Table 1).

The median concentration of TIMP-1 and TIMP-3 was lower, while TIMP-2 and TIMP-4 were higher in the study group compared to the control group. The greatest differences in concentrations were observed in TIMP-1 and TIMP-2.

In the study group, 166 eyes were analyzed. Clinical keratoconus was diagnosed in 134 eyes and subclinical keratoconus in 32 companion eyes. The severity of keratoconus was assessed based on the Amsler–Krumeich classification. Most eyes with keratoconus showed the first or second degree of disease advancement(Table 2).

**Figure 1:**
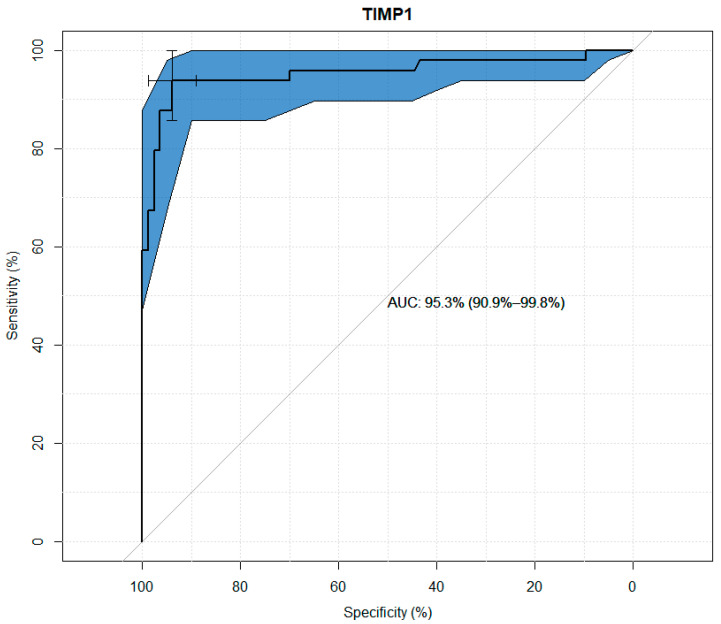
ROC curve plot for TIMP-1.

**Figure 2:**
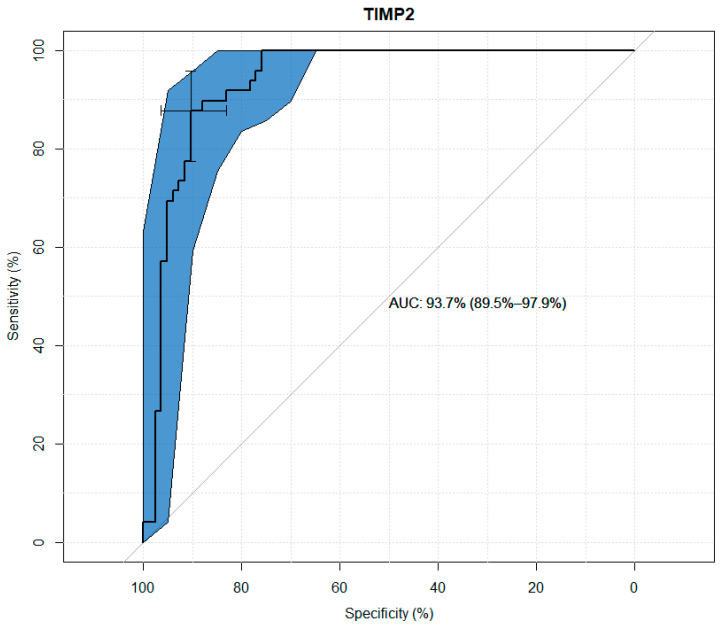
ROC curve plot for TIMP-2.

**Figure 3:**
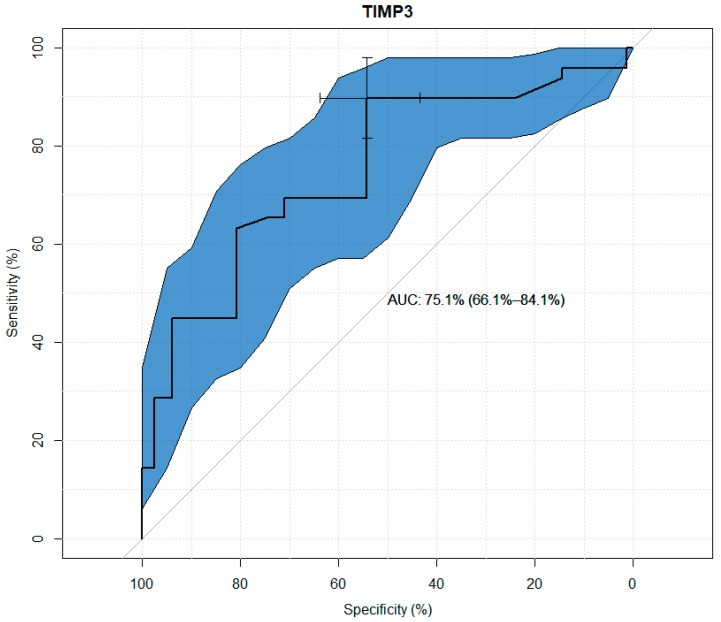
ROC curve plot for TIMP-3.

**Figure 4:**
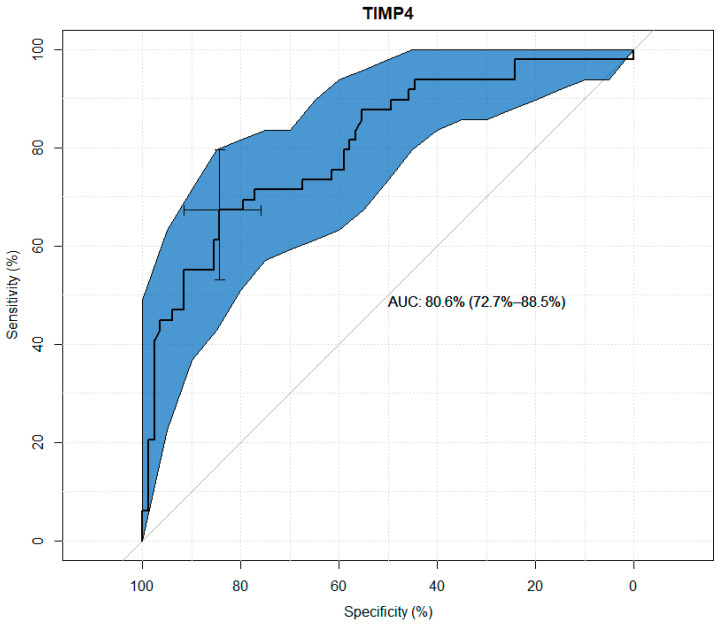
ROC curve plot for TIMP-4.

TIMP-1, TIMP-2, TIMP-3, and TIMP-4 values were analyzed for their usefulness as potential predictors of keratoconus. Significant differences were observed between the study and control groups in terms of the concentrations of all TIMPs. Through the use ofageneralized linear model, high sensitivity and specificity results were obtained for all tested variables, as presented in the figures below. The modeling results for TIMP-1 and TIMP-2 showed higher suitability as markers than the results for TIMP-3 and TIMP-4. (Figure 1, Figure 2, Figure 3 and Figure 4 and Table 3, Table 4, Table 5 and Table 6).

**Figure 5:**
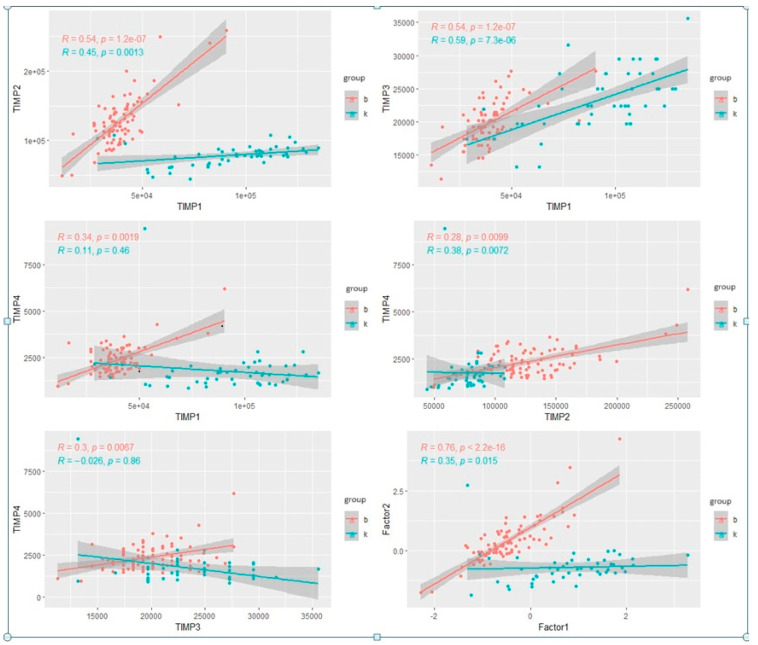
Charts of intercorrelations between individual TIMPs and the first and second factors; b = study group, c = control group.

The next step was to conduct multifactor modeling. In order to avoid the occurrence of multicollinearity, we started with the analysis of correlations between variables divided into groups (Figure 5.)

**Figure 6:**
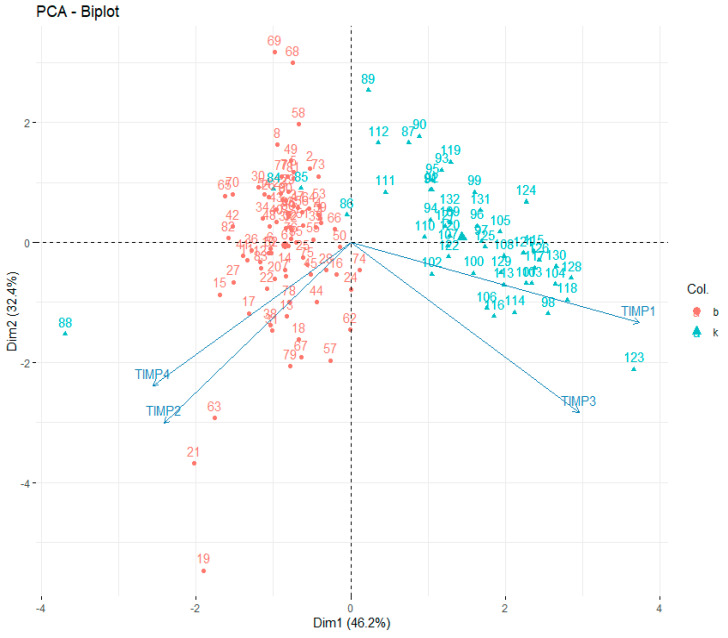
Results of principal component analysis (PCA)—biplot; b= study group, c= control group.

All TIMPs were significantly correlated with each other. Moreover, the graphical interpretation of the charts indicated the existence of certain clusters of results that seemed to clearly distinguish the control group from the study group. Due to significant correlations between the parameters used, a principal component analysis was performed to reduce the number of factors used in the analysis (Figure 6).

Two factors were obtained, explaining thetotal of 78% of variance. Factorloads for TIMP-1–4 and the values of explained variance are presented in Table 7.

The first factor correlated significantly with TIMP-1 and TIMP-3 values, while the second factor correlated significantly with TIMP-2 and TIMP-4. In order to maximize the value of explained variance, factor rotation using the varimaxnormalized method was used.

The scatterplot of the results on the plane defined by the factor values indicated the presence of clear clusters of results from the study and control groups, marked with different colors. Observation No. 88 (K5) was rejected due to being out of the set (the value of the standardized Pearson residual in the multivariate model = −374). The obtained two factors were transferred to the data set and subjected to further analysis in a generalized linear model.

**Figure 7:**
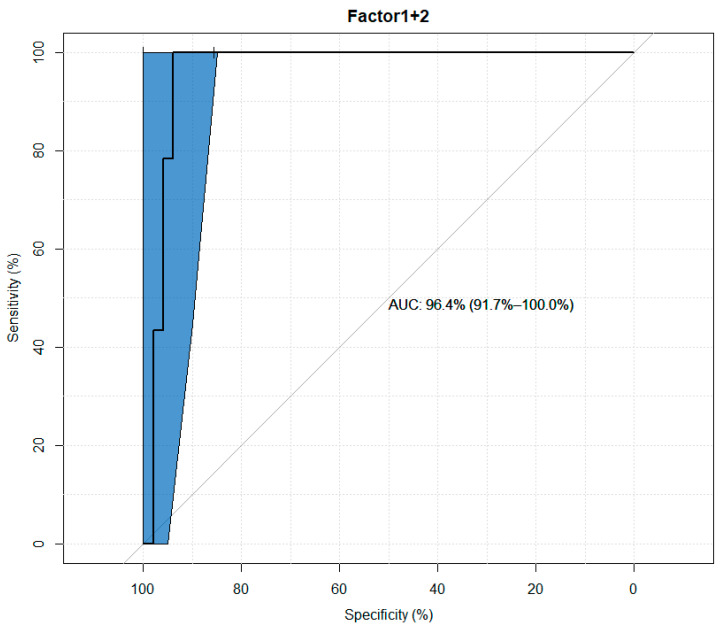
ROC curve plot for a model composed of the first and second factors.

It was observed that the model consisting of two factors obtained viaPCA analysis is characterized by better values of sensitivity, specificity and AUC than any model obtained viaunivariate analysis (Figure 7 and Table 8).

## 4. Discussion

Despite extensive basic and clinical research on keratoconus in recent years, the exact mechanisms underlying this pathology still remain unknown. The aim of this study was to evaluate selected matrix metalloproteinase inhibitors in blood serum in patients with keratoconus (KC).

Tissue metalloproteinase inhibitors, in particular TIMP-1 and TIMP-2, independently of the inhibition of matrix metalloproteinases, act as signaling molecules with cytokine-like activity, thereby influencing various biological processes, including cell growth, apoptosis, differentiation, angiogenesis, and oncogenesis. Less data areavailable on TIMP-3 and TIMP-4 in terms of cellular effects induced by direct signaling [6].

### 4.1. Tissue Inhibitor of Metalloproteinases 1 (TIMP-1)

Tissue inhibitor of metalloproteinases 1, which is a strong inhibitor of many MMPs (except some of the membrane types), is an inhibitor of collagenase, present in human serum, synthesized by monocytes, macrophages, mesenchymal cells, platelets, granulocytes and endothelial cells [7].

In histological examinations of normal corneas, TIMP-1 expression was detected in epithelial and endothelial cells, as well as in smaller amounts in stromal keratocytes, while increased TIMP-1 expression of stromal keratocytes was found in scarred keratoconus corneas [17]. A study performed by Brown et al. showed that reactive nitrogen species can reduce the production of TIMP-1 by KC corneal fibroblasts themselves [18]. Genetic studies on TIMP-1 expression have shown reduced expression in the corneal tissue of patients with keratoconus [19,20,21].

TIMP-1 and TIMP-2 have been shown to have growth-promoting properties in many different cell types, including keratocytes, fibroblasts, chondrocytes and epithelial cells. The stimulatory effect of TIMP-1 and TIMP-2 on cell proliferation is independent of its ability to inhibit MMPs. Moreover, the stimulation of cell division was found to require free non-MMP-bound TIMP-1 as its growth-promoting activity was abolished by its complex formation with proMMP or active MMPs. These findings strongly suggest that the effect of TIMP-1 on cell growth is most likely mediated by binding to the cell surface through a cell receptor mechanism. TIMP-1 binding to the cell surface does not compete with other TIMPs, especially TIMP-2, suggesting that they have their own specific receptors that are not yet fully understood [7].

Based on a study conducted on mice by Kernacki et al., it can be concluded that of all TIMPs, TIMP-1 is mainly involved in the healing process of corneal wounds and protects against extensive tissue destruction [22].

TIMP-1 may reduce corneal polymorphonuclear neutrophil granulocytes (PMN)infiltration by blocking the shedding of the adhesion molecule L-selectin present on activated PMN [23]. In addition to its effect on cell growth, TIMP-1 inhibits the process of programmed cell death, i.e., apoptosis, which occurs independently of MMPs inhibition [24].

The presented study shows that the median TIMP-1 concentration in blood serum in patients with keratoconus was more than 2.5 times reduced compared to the control group. Analyzing the above, TIMP-1 may regulate cell survival directly through activation of the appropriate receptor and through indirect mechanisms based on the inhibition of metalloproteinases. Decreased TIMP-1 values not only in corneal tissue but also in blood serum may presumably influence the intensified action of MMPs and, through their action independent of MMP inhibition, influence the intensification of keratocyte apoptosis and reduced cell proliferation, which leads to matrix degradation observed in keratoconus.

Statistical univariate analysis of TIMP-1 for usefulness as a potential predictor of keratoconus showed its sensitivity of 94% and specificity of 90% which demonstrates the strong involvement of TIMP-1 in the development of keratoconus.

### 4.2. Tissue Inhibitor of Metalloproteinases 2 (TIMP-2)

Tissue inhibitor of metalloproteinases 2 (TIMP-2) is a protein that is probably of key importance in many physiological and pathological processes, such as morphogenesis and tissue remodeling. TIMP-2 is constitutively expressed at high levels in all tissues, whereas the other three TIMPs show more selective tissue distribution patterns and can be induced by various growth factors and cytokines [7].

In immunohistochemical studies, stromal scars in keratoconic corneas had increased staining with TIMP-2 antibodies, suggesting its importance in scar formation and corneal remodeling [17]. A similar model of the association of TIMP-2 with scar formation was shown by comparing preparations of normal skin without inflammatory lesions in acne patients with a tendency toward scarring and without a tendency toward scar formation. Significantly increased expression of TLR-4, IL-2, IL-10, and TIMP-2 and decreased MMP-9 havebeen demonstrated in patients with acne prone to scarring [25]. TIMP-2 may therefore be a predictive factor of the tendency toward scar formation.Further research would be needed in this direction, especially in the context of keratoconus and the correlation of TIMP-2 concentration in the corneal tissue and its healing after a corneal cross-linking procedure.

In the presented study, the median serum TIMP-2 concentration in keratoconus patients was 1.5 times higher than in the control group. Statistical univariate analysis of TIMP-2 for its usefulness as a potential predictor of keratoconus showed its sensitivity of 88% and specificity of 90%. In summary, TIMP-2 has pleiotropic, complex functions that depend on interactions with other extracellular components, especially MMPs, as well as direct interactions with cell surface binding receptors [26]. Due to the very small number of studies on TIMP-2 in keratoconus, it is difficult to precisely determine its significance in the pathophysiology of this disease. Further research is needed to verify the above results.

### 4.3. Tissue Inhibitor of Metalloproteinases 3 (TIMP-3)

Among the four TIMPs, the tissue inhibitor of metalloproteinases 3 stands out because it is the only one with high affinity for proteoglycans in the extracellular matrix, as well as with the broadest range of inhibition, covering all MMPs and several members of the ADAM and ADAMTS families.

Matthews et al. showed an increased number of stromal cells that produce TIMP-3 in scarred keratoconus corneas and that the content of soluble TIMP-3 was significantly higher than in normal and unscarred keratoconus corneas.TIMP-3 is an MMP inhibitor typically found in association with cell matrices. However, it is unknown whether, in this state, it acts as an inhibitory MMP ligand or protects the matrix from degradation by MMPs [27].

Numerous genetic studies concerning the expression of the TIMP-3 gene have been conducted. Many of them showed a reduction in the expression of the TIMP-3 gene in patients with KC, while others did not find any mutations or any new polymorphisms for this gene [20,21,28,29]. The role of this gene is to control the balance between the destruction and the regeneration of corneal tissue by inhibiting the action of matrix metalloproteinases to protect tissues from irreversible destruction and inhibit angiogenesis. Because TIMP-3 expression was reduced in keratoconic relative to normal cornea, it was suggested that reduced expression of this gene caused an imbalance between the destruction and re-formation of interstitial substances [30]. The presented study showed lower TIMP-3 expression in blood serum in the study group than in the control group which may be consistent with the above studies. Statistical univariate analysis of TIMP-3 for its usefulness as a potential predictor of keratoconus showed itssensitivity of 54% and specificity of almost 90% which probably indicates its smaller importance in the etiopathogenesis of keratoconus compared to TIMP-1 and TIMP-2.

TIMP-3 has been attributed to pro-apoptotic properties. A study conducted by Matthews et al. showed that TIMP-3 induces the apoptosis of corneal stromal cells, while the anti-apoptotic properties of TIMP-1 protect against the apoptosis of these cells [27]. This phenomenon occurs when TIMP-3 is secreted in high concentrations into the intercellular matrix, leading to the apoptosis of cells in the vicinity, which, according to the authors of the above works, confirms the view that matrix-bound TIMP-3, and not its soluble form, causes cell death [31].

### 4.4. Tissue Inhibitor of Metalloproteinases 4 (TIMP-4)

Tissue inhibitor of metalloproteinases 4 (TIMP-4), which is a close homolog of TIMP-2, probably has similar functional properties due to its similar structure, similar enzymatic kinetics and high binding affinity to MMP-2.

In the context of keratoconus, the expression of TIMP-4 has not been studied so far, but its expression has been determined within corneal tissue and has been shown to be expressed within the epithelium, fibroblasts and endothelium, which may play a role in the modulation of extracellular matrix remodeling in corneal wound healing. TIMP-4 has been attributed antiangiogenic properties. Moreover, increased TIMP-4 immunoreactivity was observed in the course of corneal neovascularization, which was correlated with the size of vascular ingrowth [32].

In a study performed by Abu El-Asrar et al., of all four human TIMPs, TIMP-1 and TIMP-4 were found to be significantly increased in the vitreous fluid ofpatients with proliferative diabetic retinopathy (PDR). Moreover, the concentration of pro-angiogenic TIMP-1 in the vitreous humor was higher in eyes with active neovascularization in the course of PDR compared to those without active proliferation. Significant positive correlations were also found between vitreous levels of TIMP-1, TIMP-4 and MMP-9 and the key angiogenic biomarker in PDR—vascular endothelial growth factor (VEGF). The authors suggested that increased TIMP-4 levels in vitreous fluid in PDR patients, particularly those with active neovascularization, may reflect a specific regulatory mechanism enacted by the ocular microenvironment of PDR patients, possibly to counteract the angiogenic activity of TIMP-1 [33]. In contrast to the pro-angiogenic TIMP-1, several studies have provided evidence that TIMP-4 is an inhibitor of capillary endothelial cell migration but not proliferation or angiogenesis in vivo, suggesting that TIMP-4 is an important component of the negative control mechanisms of angiogenesis [34].

In the study referred to, the median TIMP-4 concentration in the study group was statistically significantly higher than in the control group. Statistical univariate analysis of TIMP-4 for its usefulness as a potential predictor of cone occurrence showed its sensitivity of 84% and specificity of 67%. Many authors do not perceive keratoconus as an inflammatory disease due to the lack of corneal neovascularization. It can therefore be assumed that increased TIMP-4 expression may be responsible for these antiangiogenic properties. In the present study, of all TIMPs, the serum TIMP-4 concentration in both patient groups was the lowest, which is consistent with other studies that have shown that TIMP-4 expression is either absent or present at a very low level in most patients tissues [35]. Due to the lack of research on TIMP-4 in keratoconus, further development of the topic in this direction is needed.

In the principal component analysis (PCA), two factors explaining a total of 78% of variance (variability naturally occurring within the parameters) were obtained, of which the first factor correlated well with the values of TIMP-1 and TIMP-3, while the second factor correlated significantly with TIMP-2 and TIMP-4, pointing to the existence of similarities between the behavior of the correlated TIMP-1 and TIMP-3, distinguishing them from TIMP-2 and TIMP-4. The values of the two obtained main components were further analyzed in a generalized linear model. This decision resulted from the existence of strong correlations between TIMP values, which would translate into the occurrence of a multicollinearity phenomenon in a generalized linear model after using them in theirnative form. The factors obtained in the PCA method are characterized by orthogonability, which allows us to eliminate the multicollinearity phenomenon, while reducing the number of elements in the model from fourtotwomakes its easier forinterpretation and in obtaining reliable results in a smaller number of cases in the base. It has been observed that the model consisting of two factors obtained in the PCA is characterized by better values of sensitivity, specificity and AUC than any model obtained viaunivariate analysis. This shows that the interaction of all TIMPs might be of essence in the etiopathogenesis of keratoconus.

## 5. Conclusions

The present study showed that the concentrations of all analyzed tissue metalloproteinase inhibitors in blood serum differed significantly between the study group and the control group, which may suggest systemic disturbances in the expression of tissue metalloproteinase inhibitors. High diagnostic sensitivity and specificity values of TIMP-1 and TIMP-2 and high diagnostic specificity values of TIMP-3 and TIMP-4 in relation to keratoconus were demonstrated, which confirms their strong role in the etiopathogenesis of this disease, especially when they all interact together.

The limitation of the study consists in the fact that the severity of keratoconus according to the Amsler–Krumeich classification among patients from the study group was at a low stage(first or second degree of disease advancement) and the analyzed patients included only stable cases. It would seem beneficial to compare these results with more advanced forms and with progressive keratoconus.

Demonstrating dysregulation in the expression of tissue inhibitors of matrix metalloproteinases in the pathogenesis of keratoconus opens the way for further research on drugs modulating systemic and/or corneal and tear film related to TIMP levels, which could slow keratoconus progression and severity.

## Figures and Tables

**Table 1:** TIMP-1, TIMP-2, TIMP-3, and TIMP-4 concentrations in the study and control group. Data are presented as the median value and the value of the first (Q1) and third (Q3) quartiles, *n*: group size, *p*: significance level.

TIMP Concentration (pg/mL)	Study Group *n* = 83			Control Group *n* = 49			
	Median Value	Q1	Q3	Median Value	Q1	Q3	*p*
TIMP-1	37,556.4	34,269.3	43,032.5	99,499.87	77,279.49	111,891.4	<0.01
TIMP-2	122,030.5	109,595.7	145,609.8	79,118.03	68,169.16	87,143.1	<0.01
TIMP-3	19,246.8	17,866.9	21,902.2	22,425.83	19,692.72	27,296.4	<0.01
TIMP-4	2210.2	1846.0	2796.5	1562.49	1185.66	1984.8	<0.01

**Table 2:** The severity of keratoconus according to the Amsler–Krumeichclassification among patients from the study group.

The Severity of KC according to the Amsler-Krumeich Classification	Number of Eyes with KC *n* = 134
I	67 (50%)
II	33 (24.6%)
III	25 (18.7%)
IV	9 (6.7%)

**Table 3:** Usefulness indicators of TIMP-1 as a marker in the diagnosis of keratoconus.

Threshold (pg/mL)	Specificity (%)	Sensitivity (%)	Negative Predictive Value (%)	Positive Predictive Value (%)
28,995.83	90	93.88	96.14	84.71

**Table 4:** Usefulness indicators of TIMP-2 as a marker in the diagnosis of keratoconus.

Threshold (pg/mL)	Specificity (%)	Sensitivity (%)	Negative Predictive Value (%)	Positive Predictive Value (%)
96,299.29	90.36	87.75	92.56	83.82

**Table 5:** Usefulness indicators of TIMP-3 as a marker in the diagnosis of keratoconus.

Threshold (pg/mL)	Specificity (%)	Sensitivity (%)	Negative Predictive Value (%)	Positive Predictive Value (%)
19,469.77	54.22	89.8	73.45	72.6

**Table 6:** Usefulness indicators of TIMP-4 as a marker in the diagnosis of keratoconus.

Threshold (pg/mL)	Specificity (%)	Sensitivity (%)	Negative Predictive Value (%)	Positive Predictive Value (%)
1692.49	84.34	67.35	77.25	76.49

**Table 7:** Factorloads for TIMP-1–4 and the value of explained variance.

	Eigenvalue	Value of Explained Variance (%)	TIMP-1	TIMP-2	TIMP-3	TIMP-4
Factor 1	1.85	46.22	**0.86**	−0.01	**0.93**	−0.12
Factor 2	1.29	32.35	−0.29	**0.89**	0.09	**0.79**

**Table 8:** Indicators of the suitability of a model composed of the first and second factors as a marker in the diagnosis of keratoconus.

Specificity (%)	Sensitivity (%)	Negative Predictive Value (%)	Positive Predictive Value (%)
90	100	100	94.42

## Data Availability

The data presented in this study are available on request from the corresponding author.
